# Theoretical Prediction of the Impact of Phosphorus Doping on the Elastic Constants of Silicon

**DOI:** 10.3390/mi16070748

**Published:** 2025-06-25

**Authors:** Azadeh Jafari, Behraad Bahreyni

**Affiliations:** Faculty of Applied Sciences, Simon Fraser University, Surrey, BC V3T0A3, Canada

**Keywords:** silicon, elastic constants, deformation potential, doping effects, MEMS

## Abstract

Accurately controlling the mechanical properties of silicon is essential for developing high-performance micro-devices and systems. In this study, we investigate the influence of phosphorus doping on the elastic constants of silicon across a wide temperature range using a combination of tight-binding simulations and deformation potential theory. The mechanical properties were derived using Keyes’s framework integrated with Fermi–Dirac statistics. The Goodwin–Skinner–Pettifor functional form was applied to estimate dopant-induced stress potentials and their effect on lattice stiffness. In particular, we investigated the change in elastic constants and their temperature dependence under ultra-high doping concentrations. The results show a monotonic decrease in c_11_ and a non-monotonic increase in c_12_ with both temperature and doping, while c_44_ remains relatively unaffected, consistent with experimental and theoretical studies. These changes are attributed to anisotropic carrier redistribution among conduction band valleys and strain-modulated interactions between valleys. The novelty of this work lies in the explicit, atomistically informed calculation of deformation potential constants using tight-binding parameters specific to phosphorus doping in silicon, enabling the accurate prediction of temperature-dependent elastic constants and anisotropic mechanical behaviour in emerging microsystem applications.

## 1. Introduction

Silicon, in both monocrystalline and polycrystalline forms, remains the backbone of the semiconductor industry, powering technologies from integrated circuits and photovoltaics to microelectromechanical systems (MEMS). While its electronic properties are well understood, an improved understanding of the mechanical behaviour of silicon under different conditions has become increasingly critical in MEMS applications for improved performance, as in gravimeters, and to meet stringent performance requirements, as in timing applications [1,2,3,4]. Understanding and engineering the elastic constants of silicon is essential for enhancing mechanical accuracy and thermal reliability. Intrinsic silicon exhibits well-characterized, temperature-dependent stiffness coefficients [5,6,7], but dopant incorporation, particularly phosphorus for n-type conductivity, introduces significant mechanical changes. These include modifications in the Young’s modulus, residual stress, and thermal expansion, all of which influence device performance [8,9,10,11]. Such dopant-induced mechanical effects are particularly relevant in MEMS, where even minor shifts in stiffness can impact sensitivity, noise, and temperature sensitivity [12,13]. Heavily doped single-crystal silicon has emerged as a promising structural material to develop microsystems that intrinsically exhibit lower temperature sensitivity, whereby tailoring doping concentrations, researchers aim to mitigate temperature-induced elastic variations and enhance MEMS device stability [14,15]. Among various dopants explored for this purpose, phosphorus stands out as the most widely used n-type dopant for silicon in microelectronics and MEMS fabrication due to its excellent activation efficiency, compatibility with CMOS processes, and predictable diffusion behaviour. Compared to other n-type dopants, such as arsenic and antimony, it offers the optimal balance between diffusivity and electrical activity, making it the most practical and experimentally validated choice for temperature-sensitive mechanical tuning in silicon-based devices [16]. 

Experimental studies [14,15,16,17,18] have demonstrated that heavy phosphorus doping leads to direction-dependent lattice softening, characterized by a reduced Young’s modulus and altered shear stiffness. These changes are primarily attributed to the anisotropic redistribution of free carriers among the conduction band valleys under strain, an effect not captured by traditional continuum elasticity models. Continuum-level approaches, such as linear elasticity theory and Voigt–Reuss–Hill averaging, have been widely used to model the mechanical behaviour of monocrystalline silicon in both bulk and microscale structures [19]. While effective for undoped or lightly doped silicon, these models assume a homogeneous, isotropic (or mildly anisotropic) medium and fail to account for localized lattice distortions, anisotropic carrier redistribution, and dopant-induced modifications to the band structure. Specifically, they neglect the electronic contributions to mechanical stiffness, strain-sensitive changes in valley degeneracy, and orbital-level interactions.

Works such as those by Bourgeois et al. [20] and Jaakkola et al. [14] relied on classical stiffness extrapolation without resolving the microscopic origin of doping effects. To overcome these limitations, we propose a multiscale modelling framework that integrates tight-binding electronic structure calculations with deformation potential theory. Our model explicitly incorporates orbital interactions between Si and P atoms—including s–p and p–p hybridizations—using dopant-specific Slater–Koster parameters fitted to ab initio band structure data. These values are derived from first-principles calculations based on the Perdew–Wang LDA formulation and norm-conserving pseudopotentials, enabling accurate representation of orbital-specific hybridization effects and electronic redistribution under strain [21,22].

A central feature of our approach is the explicit calculation of the site-specific deformation potential, which varies with dopant type and position. This is achieved through second-quantized perturbation theory that accounts for dopant-induced site energies and their spatial decay, thereby modelling the localized potential landscape of phosphorus-doped silicon. This formalism bridges the quantum mechanical Hamiltonian framework with the mechanical deformation response of the material. Unlike empirical or continuum models that assume isotropic or constant potential coefficients, our atomistically informed method captures anisotropic carrier redistribution, strain-dependent valley interactions, and directionally sensitive elastic responses. The hybrid sp^3^-based tight-binding Hamiltonian combined with deformation potential theory enables the modelling of substitutional dopant effects, valley degeneracy splitting, and localized bonding perturbations inaccessible to classical approaches. However, the model assumes ideal substitutional doping. It does not currently account for dopant clustering, defect formation, or thermal deactivation effects, which may impact accuracy at very high doping levels or elevated temperatures. This study presents a physically grounded, predictive model for computing doping- and temperature-dependent elastic constants c_11_, c_12_, and c_44_, as well as Young’s modulus and Poisson’s ratio, offering insights for the design of temperature-stable microsystems from degenerately phosphorus-doped silicon. The work also lays the foundation for studying the effect of other common and uncommon dopants to tune the mechanical properties of doped silicon.

## 2. Theoretical Model

The *N_i_* dopant atoms are randomly distributed by assigning them to randomly selected atomic sites within the silicon lattice (treated as matrix elements in the model). These substitutions are incorporated into the model as site-specific perturbations to the system’s Hamiltonian. The total tight-binding Hamiltonian of the doped silicon system is then expressed as [23,24]:

*H_Si-P_* = *H*(*k*) + H (1)

where *H*(*k*) represents the electronic structure of undoped (pure) silicon in momentum space, and *H* accounts for the perturbation introduced by the dopant atoms.

The average energy contribution of the dopants is given by *E_i_* = ⟨*H_Si-P_*⟩ = ⟨*H*(*k*)⟩ + ⟨*H*⟩. This represents the potential strength induced by doping.

This formulation is grounded in quantum mechanical principles, where Hamiltonian operators and their expectation values are used to describe the system’s total energy [25,26]. By decomposing the Hamiltonian into contributions from the pristine lattice and dopant-induced local perturbations, the model captures both extended and localized electronic effects critical to understanding mechanical and electronic behaviour in doped semiconductors.

The expectation values are calculated using the quantum state vector ψ, a fundamental construct in wave mechanics. The average energy contribution from the unperturbed system is given by the inner product ψ^†^*H*ψ = ⟨*H*(*k*)⟩. This formulation yields a physically meaningful scalar that reflects the system’s electronic energy [27]. Widely used in theoretical solid-state physics, this approach offers atomistic insights into carrier transport, band structure modulation, and impurity scattering phenomena, particularly within the tight-binding formalism. It provides a critical link between quantum mechanical descriptions of electrons and practical device-level modelling in semiconductor engineering. In this expression, ψ is the state vector (a column of complex amplitudes), H is the Hamiltonian matrix, and ψ^†^ is the Hermitian conjugate (complex transpose) of ψ [28].

To describe the electronic structure of pure crystalline silicon in momentum space, we employ a tight-binding Hamiltonian defined for a bipartite lattice system composed of sublattices A and B. The reciprocal-space Hamiltonian takes the block matrix form [29,30,31]:

(2) $$H\left(k\right)=\left[\begin{array}{cc} H_{AA}(k) & H_{AB}(k)\\ H_{BA}(k) & H_{BB}(k) \end{array}\right]$$

In this formulation, the blocks *H_AA_* and *H_BB_* encompass the hoppings between sites within the same sublattice, while the blocks *H_AB_* and *H_BA_* = *H*^†^*_AB_* involve hoppings between sites in different sublattices [30]. The dimensions of these blocks are contingent upon the number of orbitals considered in the tight-binding model. In silicon, the blocks are 4 × 4 if one takes account of only the s, px, py, and pz orbitals (or 5 × 5 in the sp^3^s^∗^ model, including the s^∗^ orbital) [21].

Under this approximation, sites within the same sublattice are not connected, so that diagonal blocks simply contain the on-site potential energies *H_AA/BB_* = diag(Es, Ep, Ep, Ep, Es^∗^), while the inter-sublattice hoppings contain the non-trivial momentum dependence:

(3) $$H_{\mathrm{AB}}(k)=\;\left[\begin{array}{ccccc} Vssg0(k) & Vspg1(k) & Vspg2(k) & Vspg3(k) & 0\\ -Vspg1(k)\;\;\; & Vxxg0(k) & Vxyg3(k) & Vxyg2(k) & -Vs*pg1(k)\\ -Vspg2(k) & Vxyg3(k) & Vxxg0(k) & Vxyg1(k) & -Vs*pg2(k)\\ -Vspg3(k) & -Vxyg1(k) & -Vxyg1(k) & -Vxxg0(k) & -Vs*pg3(k)\\ 0 & -Vs*pg1(k) & -Vs*pg2(k) & Vs*pg3(k) & 0 \end{array}\right]$$

where momentum functions are:

(4) $$g_{0}(k)\;=\;\frac{1}{4}\;(e^{{\mathrm{id}}_{0}k}\;+\;e^{{\mathrm{id}}_{1}k}\;+\;e^{{\mathrm{id}}_{2}k}\;+\;e^{{\mathrm{id}}_{3}k}),\;g_{1}(k)\;=\;\frac{1}{4}\;(e^{{\mathrm{id}}_{0}k}\;+\;e^{{\mathrm{id}}_{1}k}\;+\;e^{{\mathrm{id}}_{2}k}\;-\;e^{{\mathrm{id}}_{3}k}),\;g_{2}(k)\;=\;\frac{1}{4}\;(e^{{\mathrm{id}}_{0}k}\;-\;e^{{\mathrm{id}}_{1}k}\;+\;e^{{\mathrm{id}}_{2}k}\;-\;e^{{\mathrm{id}}_{3}k}),\;g_{3}(k)\;=\;\frac{1}{4}\;(e^{{\mathrm{id}}_{0}k}\;-\;e^{{\mathrm{id}}_{1}k}\;-\;e^{{\mathrm{id}}_{2}k}\;+\;e^{{\mathrm{id}}_{3}k}),$$

and d_0_ = a/4(1 1 1), d_1_ = a/4 (1 −1 −1), d_2_ = a/4 (−1 1 −1), and d_3_ = a/4 (−1 −1 1), with a = 5.4310 Ǻ. The interaction parameters in the Hamiltonian are (in eV) E_s_ = −4.20, E_p_ = 1.72, E_s∗_ = 6.69, V_ss_ = −8.30, V_sp_ = 5.73, V_s∗p_ = 5.38, V_xx_ = 1.72, V_xy_ = 4.58 [32].

In Equation (3), the terms V_ss_, V_sp_, V_xx_, and V_xy_ represent orbital interaction integrals that define electron hopping amplitudes between neighbouring atomic sites. Specifically: V_ss_: s–s σ bonding integral (hopping between s-orbitals), V_sp_: s–p σ bonding (coupling between s and p orbitals along the bond axis), V_xx_: p–p σ bonding (interaction between p-orbitals aligned along the same axis), V_xy_: p–p π bonding (interaction between orthogonal p-orbitals). V_s∗p_: s*–p σ bonding (interaction between an excited s-orbital and a p-orbital along the bond axis; used in extended sp^3^s* models to improve conduction band accuracy). This matrix formalism produces a Hermitian 10 × 10 matrix *H*(*k*) that describes the full band structure of pure silicon in momentum space. The electronic state is expressed as a 10 × 1 wavefunction vector ψ(k), and the expectation value of the energy is obtained as: ⟨H(k)⟩ = ψ^†^(k)H(k)ψ(k). This scalar result provides the average energy of the system at wave vector k, allowing efficient evaluation of the electronic band structure, dispersion relations, and perturbative energy shifts. The wave function in reciprocal space is expressed as a 10 × 1 column vector. The wave function ψ(k = 0) was obtained by diagonalizing a 10 × 10 empirical tight-binding Hamiltonian constructed using the sp^3^s* basis for silicon, following the method and parameter sets introduced by Slater and Koster [21], and extended in semiconductor modelling by Jancu et al. [33]. The formulation is compatible with numerical evaluation in MATLAB R2023b cross the Brillouin zone and forms the theoretical backbone for modelling quantum transport and doping effects in semiconductor materials.

In Equation (1), the second component of the total Hamiltonian accounts for the local perturbations introduced by phosphorus dopants in the silicon lattice. This perturbative Hamiltonian is written in second quantization as [26]:

(5) $$H=\Sigma_{i}\;\varepsilon_{i}\;a_{i}^{+}a_{i}$$

Second quantization is a formalism in quantum mechanics where particles are described by creation and annihilation operators acting on quantum states, allowing for a natural treatment of many-body systems and occupation numbers where $a_{i}^{+}$ and *a_i_* are the annihilator and creator operator of the electron, respectively. In the presence of dopant atoms, *N_i_* dopant atoms are randomly distributed among *N_m_* silicon atomic sites. The potential *ɛ_i_* energy of the *i*th site at position *r_i_* is induced by these dopant atoms Goodwin et al., who recommended the following function for distance scaling [31]:

(6) $$\varepsilon_{i}(r_{i})=\sum_{n=0}^{N_{i}}V_{n}\exp\left(-\frac{\left|r_{i}-r_{n}\right|}{2d^{2}}\;\right)$$

where *V_n_* is the average of the potential strength of the dopant atoms, which we utilized from references [33,34], *r_n_* is the location of the *n*th dopant atoms, and *d* stands for the spatial range of the dopant potential (2.35 Å), which is proportional to the average distance between Si and dopant atoms. Substituting Equation (6) into Equation (5), the doped Hamiltonian becomes:

(7) $$H=\sum_{i}[\sum_{n-1}^{N_{i}}V_{n}\;exp(-\frac{\left|r_{i}-r_{n}\right|}{2d^{2}})]a_{i}^{+}\;a_{i}\;$$

This form captures the site-dependent energy modulation resulting from all dopants, reflecting the locality and decay of their electrostatic influence. To compute the average energy of the system in quantum state ∣ψ⟩, we calculate:

(8) $$\langle H\rangle =\sum_{i}[\sum_{n-1}^{N_{i}}V_{n}\;exp(-\frac{\left|r_{i}-r_{n}\right|}{2d^{2}})].{\left|\psi_{i}\right|}^{2}$$

to each site *i*, depending on their relative position. In the matrix, by using a diagonal Hamiltonian matrix, we have:

(9) $$H_{ii}=\sum_{n-1}^{N_{i}}V_{n}\;exp(-\frac{\left|r_{i}-r_{n}\right|}{2d^{2}})$$

When phosphorus is introduced into the silicon lattice as a substitutional dopant, it alters the local electronic potential landscape, affecting the standard tight-binding interactions typically defined for pure silicon. In particular, the off-diagonal elements of the Hamiltonian (*H_ij_*), which represent electron hopping between neighbouring atomic orbitals, are modified to reflect Si–P interactions. For this, we adopt a refined set of matrix elements, including *V_ssσ_*, *V_spσ_*, *V_psσ_*, *V_ppσ_*, and *V_ppπ_*. These parameters were derived from empirical tight-binding (ETB) models fitted for zincblende-type Si systems and were specifically chosen to capture changes in orbital overlap and bonding character introduced by phosphorus atoms [32]. These fitted values play a central role in determining the anisotropic, direction-dependent electronic band structure and transport properties of the doped silicon system. Parameterization was carried out based on ab initio band structure data, originally computed using a plane-wave density functional theory (DFT) approach following the Hohenberg-Kohn and Kohn-Sham formulation [35,36]. In this context, norm-conserving pseudopotentials were employed to model core-valence interactions, and the Perdew–Wang local-density approximation (LDA) was used for exchange-correlation effects. The supercell included two Si atoms and two dopant atoms, ensuring 14 valence electrons and full occupation of seven bands, thereby avoiding spin-polarization artifacts [22]. To further improve physical accuracy, we incorporate a central-cell correction (CCC) of approximately −3.5 eV, which accounts for the short-range potential well induced by the dopant [37]. Finally, we note that doping can also perturb hopping integrals due to changes in local bond lengths and lattice strain. Thus, the interaction parameters may require additional empirical or DFT-guided refinement to represent bond weakening, charge redistribution, and strain localization effects. Such adjustments, including the CCC and modified hopping integrals, are crucial for simulating realistic doped systems and have been successfully applied in studies such as those by Martins et al. [38], which demonstrated accurate reproduction of conduction-band minima and effective masses in phosphorus-doped silicon. *V_n_* represents the strength of the local potential introduced by a phosphorus dopant, accounting for short-range central-cell corrections and bonding differences with silicon. The decay constant d defines how rapidly this potential decreases with distance, reflecting the Si–P bond length and screening effects. Together, they describe a short-range exponential potential that captures the dopant’s localized influence on the host lattice.

In this study, phosphorus atoms are modelled as substitutional dopants, replacing silicon atoms at random lattice sites, an approach consistent with the dominant doping mechanism observed in n-type silicon. Interstitial doping is not considered due to its higher formation energy and less predictable electronic behaviour. All dopant-induced perturbations, including on-site energy shifts and modified hopping integrals, are based on this substitutional assumption. While the tight-binding and deformation potential-based model provides improved accuracy in capturing doping-induced elastic changes, it assumes ideal substitutional doping and does not account for complex effects such as defect formation, dopant clustering, or thermal diffusion, which may become significant at high doping levels or elevated temperatures. The Duhamel-Neumann extension of Hooke’s law to the thermoelastic constitutive connection between stress and strain is commonly used to characterize single-crystal silicon, an anisotropic media that is thought to be linear [4,10,12]:

(10) $$\sigma_{ij}=c_{ijkl}\left[\varepsilon_{kl}-\alpha_{kl}\left(T-T_{0}\right)\right]$$

where *T*_0_ is the reference temperature corresponding to the stress-free state (the ambient temperature), *T* is the temperature such that (*T* − *T*_0_)/*T*_0_ ≤ 1, the subscripts *i*, *j*, *k*, *l* = 1, 2, 3 in the Cartesian coordinate system, and *σ_ij_* and *ε_kl_* are the components of the stress and strain tensors and α_kl_ is the thermal expansion coefficient. By substituting the indices with (11 → 1, 22 → 2, 33 → 3, 23 → 4, 13 → 5, 12 → 6), the Voigt convention converts the stress and strain in Equation (11) into column vectors and the fourth-rank elasticity tensor into a 6 × 6 symmetric square matrix. Consequently, the silicon elastic tensor in Voigt’s notation takes the following form:

(11) $$C=\left(\begin{array}{cc} \begin{array}{ccc} c_{11} & c_{12} & c_{12}\\ c_{12} & c_{11} & c_{12}\\ c_{12} & c_{12} & c_{11} \end{array} & \begin{array}{ccc} 0 & 0 & 0\\ 0 & 0 & 0\\ 0 & 0 & 0 \end{array}\\ \begin{array}{ccc} 0 & 0 & 0\\ 0 & 0 & 0\\ 0 & 0 & 0 \end{array} & \begin{array}{ccc} c_{44} & 0 & 0\\ 0 & c_{44} & 0\\ 0 & 0 & c_{44} \end{array} \end{array}\right)$$

Therefore, εkl = 1/2(u_i,j_ + u_j,i_) can be used to define the elastic strain in terms of the displacement components u_i_, where u_i,j_ stands for the derivative of the displacement component with respect to the spatial variable x_j_.

An outstanding experimental effort incorporating the theoretical analysis has been presented [39,40], especially in the case of n-doped silicon. The doping-induced changes to the elastic constants at absolute temperature T are as follows [35]:

(12) $$\delta c_{11}\;=-\frac{2}{9}\left(\frac{NE_{i}^{2}}{k_{B}T}\right)\left(\frac{\overset{´}{\frac{F1}{2}}\left(\eta \right)}{\frac{F1}{2\left(\eta \right)}}\right)\;\delta c_{12}\;=\frac{1}{9}\left(\frac{NE_{i}^{2}}{k_{B}T}\right)\left(\frac{\overset{´}{\frac{F1}{2}}\left(\eta \right)}{\frac{F1}{2\left(\eta \right)}}\right)\;\delta c_{44}=\frac{1}{9}\left(\frac{NE_{i}^{2}}{k_{B}T}\right)$$

where $E_{i}$ is the potential strength due to the induced doping, *F*_1/2_ is the corresponding Fermi energy, and the total density of electrons is given by [39,40,41]:

(13) $$N\left(T,\;\eta \right)=\frac{12}{\sqrt{\pi }}N_{c}F_{\frac{1}{2}}\left(\eta \right)$$

where *Nc* is the effective density of states in the conduction band and is defined by:

(14) $$N_{c}\left(T\right)=2\left(2\pi m\frac{k_{B}T}{h^{2}}\right)3^{/2}$$

where *h* is the Plank constant and m is the density-of-states effective mass for silicon *m* = 0.324 *m_0_* with *m*_0_ defined as the mass of the electron.

In Equation (12), *η* is the reduced Fermi energy, *N* is the electron density, $k_{B}$ is the Boltzmann constant and:

(15) $$F_{k}\left(\eta \right)=\int_{0}^{\infty }\frac{t^{k}dt}{e^{t-\eta }+1}$$

is the Fermi integral of the order of k, and prime indicates its derivative with respect to *η*. The degeneracy level, which is connected to the Fermi energy level, is represented by the *η* parameter. The correlation between the free electron concentration in the conduction band and the Fermi integral is also important to observe. By taking into account extremely small strains and extending the strain-dependent portions of the Fermi integrals in terms of values proportional to the strain, the elastic constants are computed. In considering this, writing *η* as follows is convenient [39]:

*η* = *η*_0_ + *δ* (16)

where *δ* is the change in η with strain, Ef is the fermi energy, and *η_0_* is (*Ef*/*kT*) in the unstrained lattice. Thus, by integrating the earlier discussion based on Keyes’s theory with the measured elastic constants over temperature reported for intrinsic silicon, it is possible to compute the temperature dependence of the silicon elastic constants at different doping concentration levels. The tight-binding Hamiltonian governs electron hopping between atomic orbitals in the silicon lattice. When phosphorus atoms are introduced as substitutional dopants, they locally modify the site energies of nearby silicon atoms—these changes are introduced into the Hamiltonian through position-dependent potential terms. The resulting perturbations alter the electronic band structure and redistribute charge carriers. These electronic shifts affect interatomic bonding forces, particularly under mechanical strain. For example, dopants screen interatomic interactions and reduce bond stiffness in certain crystallographic directions, leading to measurable changes in elastic constants such as c_11_, c_12_, and c_44_. This coupling between electronic structure and mechanical response is captured using deformation potential theory, which relates the strain-dependent average energy shift Ei to modifications in elastic behaviour.

To calculate the temperature-dependent elastic constants *c*_11_, *c*_12_, and *c*_44_ for different phosphorus concentrations, the following relationship is used:

(17) $$c_{ij}\left(T\right)=c_{ij,pure}\left(T\right)+\delta c_{ij}\left(T\right)$$

where;

$$\frac{1}{c_{ij,pure}}\frac{{dc}_{ij,pure}}{dT}=\alpha_{ij}$$

And for {*ij*} = {11, 12, 44}:

(18) $$\alpha_{11}=-75.3\times 10^{-6}K^{-1}\;\alpha_{12}=-24.5\times 10^{-6}K^{-1}\;\alpha_{44}=-55.5\times 10^{-6}K^{-1}$$

**Figure 1:**
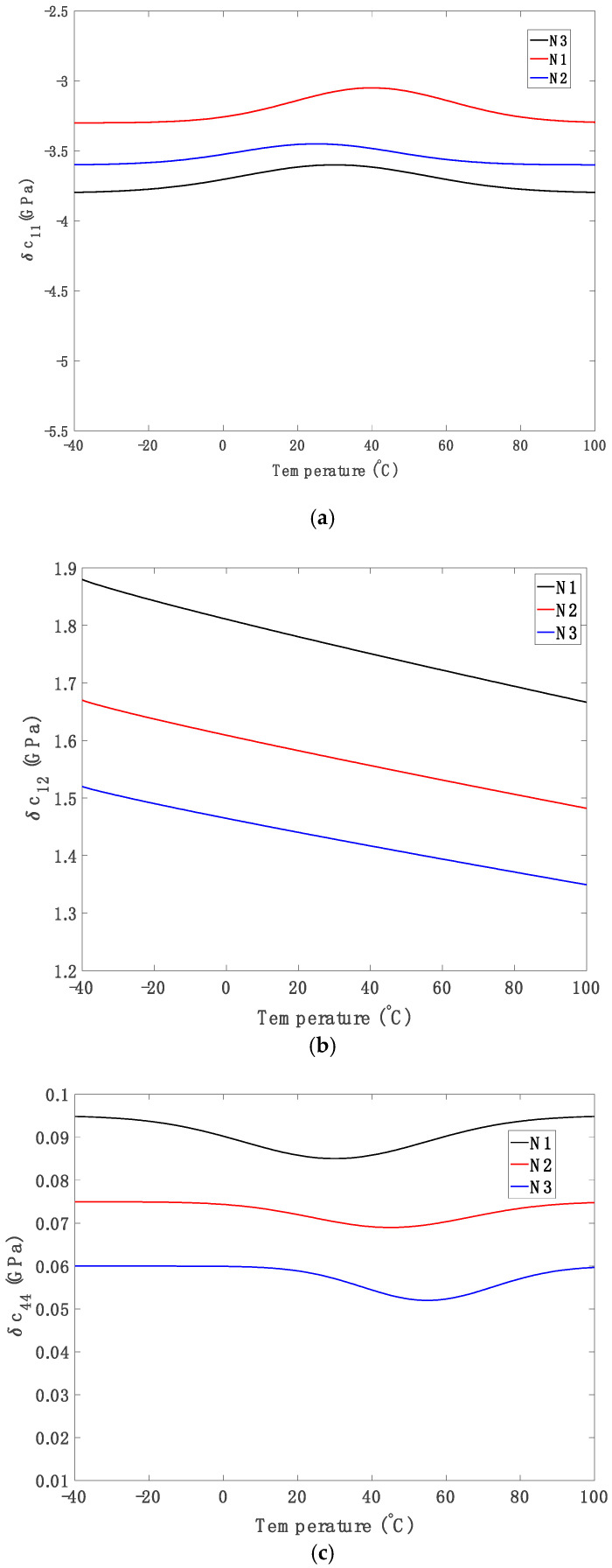
Elastic component (**a**) *δc*_11_, (**b**) *δc*_12_, (**c**) *δc*_44_ change versus temperature for phosphorus at *N*1 = 1 × 10^19^ cm^−3^, *N*2 = 3 × 10^19^ cm^−3^ and *N*3 = 6 × 10^19^ cm^−3^ concentrations in silicon.

**Figure 2:**
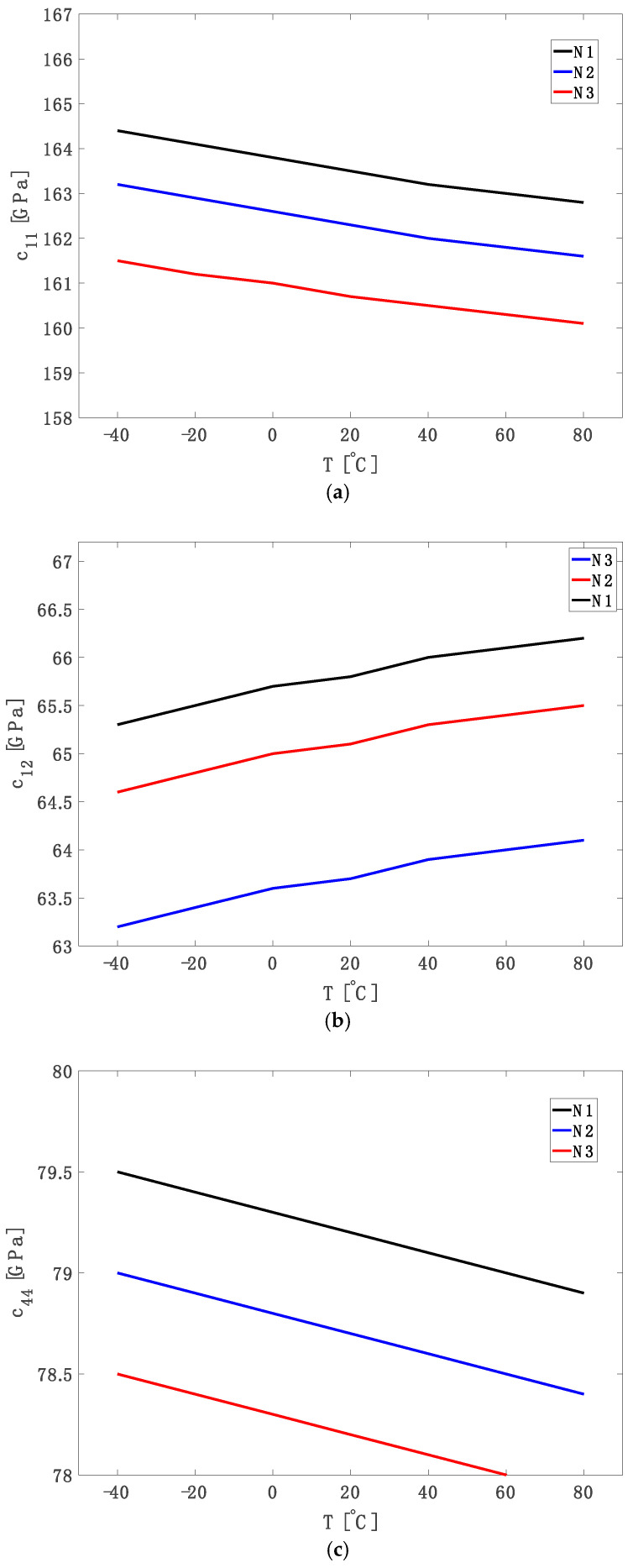
Elastic component (**a**) *c*_11_, (**b**) *c*_12_ and (**c**) *c*_44_ versus temperature for different phosphorus concentrations (*N*1 = 1 × 10^19^ cm^−3^, *N*2 = 3 × 10^19^ cm^−3^ and *N*3 = 6 × 10^19^ cm^−3^).

These coefficients are empirical values obtained by fitting experimental data on single-crystal silicon [14,42]. Also, $c_{ij,pure}\left(T\right)=c_{ij,pure}\left(T0\right).exp[\alpha_{ij}\left(T-T0\right)]$. The doping-induced changes in the elastic component, *δc_ij_*, are derived from Equation (12) and the corresponding curves are shown in Figure 1. The total elastic constants, *c_ij_*(*T*), incorporating both pure ($c_{ij,pure}\left(T\right)$) and doping effects, are shown in Figure 2.

## 3. Results and Discussion

Using Equation (12), Figure 1a–c show the temperature-dependent variations of δc_11_, δc_12_, and δc_44_ for different phosphorus doping concentrations. The three selected doping concentrations span from lightly to heavily doped regimes, remaining below the solid solubility limit of phosphorus in silicon (~1.3 × 10^21^ cm^−3^). These values align with prior studies, ensuring both relevance and verifiability of the results. The elastic constant *c*_11_ reflects stiffness under uniaxial extension, while *c*_12_ describes the transverse response to longitudinal strain in cubic crystals [42].

Figure 1a illustrates the variation of *δc*_11_ (the change in the longitudinal elastic constant c_11_) as a function of temperature for three phosphorus doping concentrations: *N*_1_ = 1 × 10^19^ cm^−3^, *N*_2_ = 3 × 10^19^ cm^−3^, and *N*_3_ = 6 × 10^19^ cm^−3^. Across the entire temperature range (−40 °C to 100 °C), δc_11_ remains negative for all doping levels, indicating a consistent softening of the longitudinal stiffness with increasing temperature due to free carrier redistribution that weakens the interatomic bonding along the ⟨100⟩ crystallographic direction.

Similarly, as shown in Figure 1b, *δc*_12_ decreases monotonically with temperature, reflecting enhanced lateral compliance. This behaviour suggests that phosphorus-doped silicon becomes increasingly flexible at elevated temperatures, likely due to temperature-induced modifications in atomic interactions and dopant effects, including the weakening of interatomic forces such as ionic or van der Waals interactions, which reduces resistance to deformation along the c_12_ axis [42].

Figure 1c presents the variation of the shear elastic constant δc_44_ with temperature for three phosphorus doping levels. Although *δc*_44_ exhibits relatively small absolute changes compared to *δc*_11_ and *δc*_12_, it shows a distinct non-linear temperature dependence, especially at higher doping concentrations (e.g., *N*_3_ = 6 × 10^19^ cm^−3^). These behaviours are attributed to the anisotropic redistribution of electrons among equivalent conduction band valleys under strain, affecting bond stiffness differently along crystallographic directions [42]. Phosphorus doping introduces additional free carriers and local lattice distortions governed by (i) silicon sp^3^ hybridization, (ii) electron donation from phosphorus 4p orbitals, and (iii) formation of modified covalent bonds with neighbouring Si atoms [41,42]. Additionally, factors such as the equilibrium distribution coefficient and solid solubility limit of phosphorus contribute to the final dopant configuration [39,40].

Using Equation (17), Figure 2a–c illustrates the absolute elastic constants *c*_11_, *c*_12_, and *c*_44_ as functions of temperature for three phosphorus doping levels (*N*1 = 1 × 10^19^ cm^−3^, *N*2 = 3 × 10^19^ cm^−3^ and *N*3 = 6 × 10^19^ cm^−3^). In Figure 2a, c_11_ decreases linearly with temperature, while higher doping levels reduce its absolute value, confirming longitudinal lattice softening. Figure 2b demonstrates a positive temperature coefficient for c_12_, more prominent at lower doping levels, suggesting enhanced transverse bond fluctuations due to thermal vibrations. Figure 2c shows that c_44_ declines uniformly with temperature and exhibits less sensitivity to doping compared to c_11_ and c_12_. These observations reflect a combination of carrier screening, dopant-induced bond weakening, and thermal effects [39,42].

**Figure 3:**
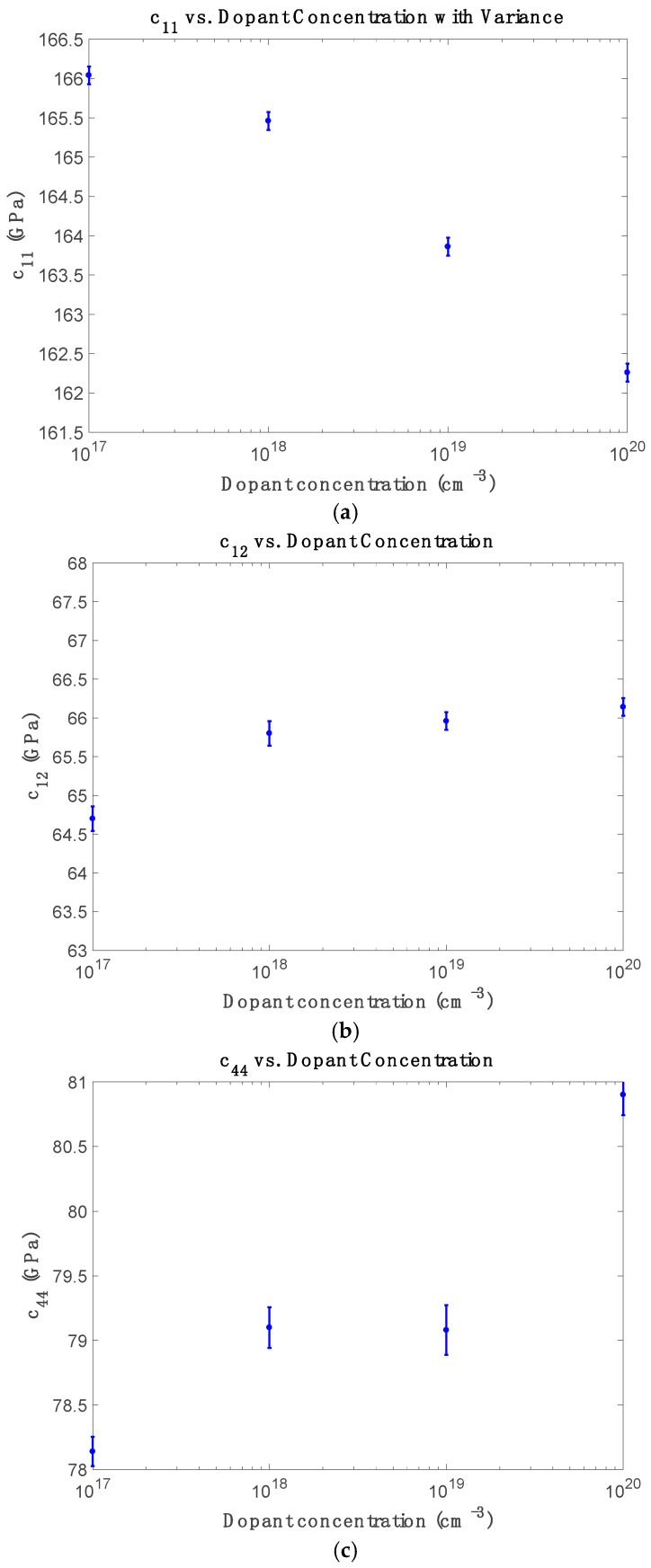
Elastic component (**a**) *c*_11_, (**b**) *c*_12_ and (**c**) *c*_44_ versus concentrations of phosphorus at room temperature.

Figure 3 displays the doping dependence of elastic constants at room temperature. Multiple independent simulations were conducted for each doping concentration to capture statistical variation by randomly distributing phosphorus atoms at substitutional lattice sites. In each realization, the dopant-induced site energy shifts and hopping modifications were applied to the tight-binding Hamiltonian to compute *c*_11_, *c*_12_, and *c*_44_. The error bars represent the standard deviation across these simulation runs, accounting for variability arising from dopant configurations and short-range interactions. As shown in Figure 3a, c_11_ decreases monotonically with increasing doping concentration, confirming progressive lattice softening due to free-carrier screening and weakened bonding. In contrast, Figure 3b shows that c_12_ increases non-linearly with doping, attributed to anisotropic charge redistribution and enhanced bond strength under transverse strain—consistent with Ng et al. [15]. Figure 3c reveals that while c_44_ remains nearly unchanged at moderate doping, it exhibits an unexpected increase at the highest concentration (10^20^ cm^−3^), possibly due to local stress pinning, clustering, or dopant-induced strain effects at high carrier densities.

Table 1 compares our simulated values of c_11_ and c_12_ against experimental data from prior studies on heavily phosphorus-doped silicon. The model shows strong agreement across all concentrations, particularly with the work of Ng et al. [15], Jaakkola et al. [10,14], and Bourgeois et al. [20]. For instance, at 6 × 10^19^ cm^−3^, our results deviate by less than 2.5% from the experimental data, confirming the model’s predictive accuracy.

**Figure 4:**
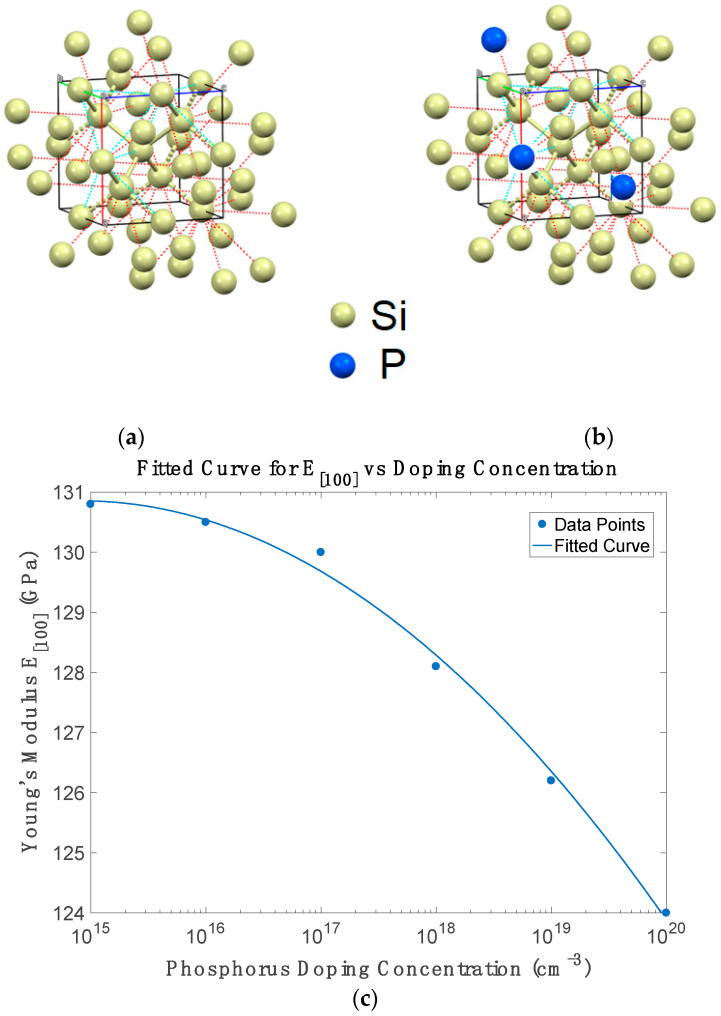

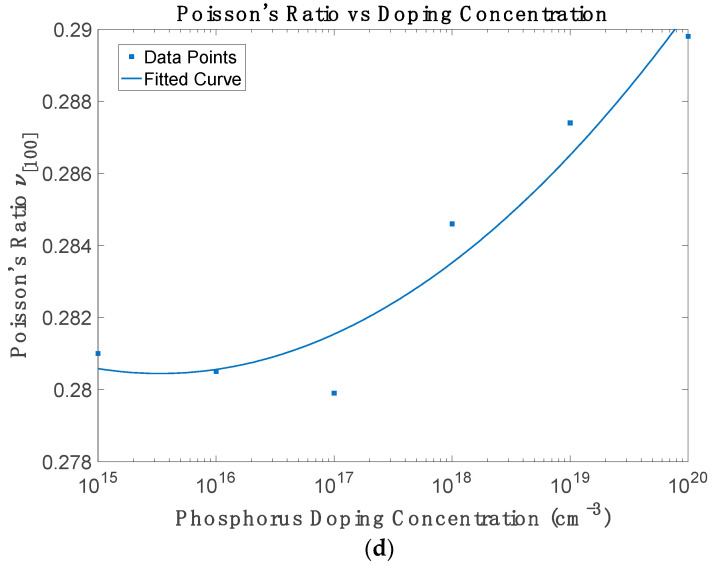
(**a**,**b**) Lattice structure of silicon before and after phosphorous doping (**c**) Young’s modulus and (**d**) Poisson’s ratio of phosphorus-doped silicon as a function of doping concentration at room temperature.

For cubic silicon crystals, the direction-dependent Young’s modulus and Poisson’s ratio can be derived from the elastic stiffness constants c_11_ and c_12_ [14,16]. Specifically, for the crystallographic [100] direction, these quantities are expressed as: E[100] = (c_11_ + 2c_12_)(c_11_ − c_12_)/(c_11_ + c_12_) and ν[100] = c_12_/(c_11_ + c_12_). Figure 4c shows that Young’s modulus decreases nearly linearly with doping concentration from 1 × 10^15^ to 1 × 10^20^ cm^−3^, consistent with free-carrier screening effects that weaken covalent bonds under strain. This result agrees with experimental findings by Zeng et al. and Ng et al. [15,16]. Additionally, our predicted variation aligns closely with Vanhellemont et al. [44], who reported Young’s modulus decreasing from ~130.0 GPa in undoped silicon to ~127.3 GPa at 2.9 × 10^19^ cm^−3^. Similarly, ab initio simulations by Kamiyama and Sueoka [45] confirm the softening trend via CASTEP supercell modelling and Vegard’s law interpolation.

Figure 4d shows that Poisson’s ratio increases with doping concentration, reflecting increased lateral compliance under uniaxial stress. This trend results from the smaller reduction in c_12_ compared to c_11_, thus enhancing transverse strain relative to axial strain. These changes are critical for MEMS devices, where both stiffness and transverse compliance affect resonator stability and temperature compensation [14,15]. The variation in Poisson’s ratio with phosphorus doping exhibits a non-monotonic trend. At low doping concentrations (up to approximately 10^17^ cm^−31^), Poisson’s ratio slightly decreases as c_12_ decreases more rapidly than c_11_, reflecting a modest reduction in lateral compliance due to localized bond distortions. Beyond 10^17^ cm^−31^, the ratio increases steadily with doping as enhanced electronic screening and carrier-induced bond softening begin to dominate, reducing c_11_ more significantly than c_12_. This leads to increased lateral deformation under uniaxial stress. The overall behaviour captures two competing effects: initial stiffening from isolated impurity atoms, followed by softening at higher doping due to free-carrier interactions with the lattice. This trend aligns with theoretical expectations involving valley redistribution, charge screening, and lattice relaxation and is consistent with prior studies [15,44]. At very high doping levels, additional contributions such as dopant clustering and strain accumulation may further enhance the lateral compliance of the material [45,46]. Furthermore, the deviation from strict linearity at moderate concentrations (around 10^18^ to 10^19^ cm^−3^) could indicate intermediate regimes of dopant-induced clustering, local strain effects, or non-uniform carrier redistribution, which are not fully captured in continuum approximations. This underscores the value of atomistic models, such as the one used here, for revealing subtle nonlinearities in mechanical behaviour.

## 4. Conclusions

This study introduces a multiscale modelling framework that integrates tight-binding electronic structure with deformation potential theory to investigate how phosphorus doping modifies the elastic properties of silicon, with relevance for thermally stable MEMS applications. In contrast to earlier continuum-based or empirical models, our approach incorporates dopant-specific orbital interactions using the Goodwin–Skinner–Pettifor formalism, enabling direct calculation of strain-dependent elastic constants. Our results show that phosphorus doping reduces longitudinal stiffness (c_11_), slightly increases transverse stiffness (c_12_), and modestly affects shear (c_44_). The softening of the lattice arises from carrier redistribution among conduction band valleys while doping also reduces the temperature sensitivity of elastic constants, offering intrinsic thermal compensation for MEMS resonators. Young’s modulus decreases, and Poisson’s ratio slightly increases with doping, which is consistent with free-carrier screening effects. While the proposed framework offers improved accuracy through tight-binding and deformation potential theory, it relies on several assumptions that introduce limitations. These include the assumption of ideal substitutional doping, neglect of dopant clustering, thermal diffusion, and vacancy defect formation, as well as the use of empirical pTablearameter fitting, which may limit transferability to other dopants or materials. Additionally, the model currently omits phonon coupling, which could influence elastic properties under certain conditions. Future work will extend the model to other dopants and include defect and thermal effects using atomistic simulations. Coupling with FEM and experimental validation will improve its relevance for MEMS applications.

## Figures and Tables

**Table 1:** A comparison between predictions by our model and previously reported values of linear elastic constants (all reported in GPa) at room temperature.

Dopant Concentration	C_11_	C_12_	C_11_	C_12_	C_11_	C_12_	C_11_	C_12_	C_11_	C_12_
6 × 10^19^ cm^−3^	161.41	63.7	164	66.7					164	66.7
1 × 10^19^ cm^−3^	164.96	65.5			163.94	64.77			163.94	64.77
3 × 10^19^ cm^−3^	162.76	65.1					163	65.4		
Reference	This work	This work	[14]	[14]	[15]	[15]	[20]	[20]	[43]	[43]

## Data Availability

The original contributions presented in this study are included in the article. Further inquiries can be directed to the corresponding author.
